# Liquiritin Alleviates Depression-Like Behavior in CUMS Mice by Inhibiting Oxidative Stress and NLRP3 Inflammasome in Hippocampus

**DOI:** 10.1155/2022/7558825

**Published:** 2022-01-11

**Authors:** Chang Liu, Dai Yuan, Chi Zhang, Ye Tao, Ying Meng, Mengli Jin, Wu Song, Bingmei Wang, Lin Wei

**Affiliations:** ^1^Clinical Medical College of Changchun University of Chinese Medicine, Changchun 130117, China; ^2^School of Basic Medicine, Changchun University of Chinese Medicine, Changchun 130117, China; ^3^Affiliated Hospital of Changchun University of Chinese Medicine, Changchun 130021, China

## Abstract

**Objective:**

Central inflammation is generally accepted to be involved in the pathology of depression. We investigated whether liquiritin exerts antidepressant effects by inhibiting central NLRP3 inflammasomes.

**Results:**

The behavioral despair model and chronic unpredictable mild stress (CUMS) model in mice were established to evaluate the antidepressant action of liquiritin. In the despair model study, liquiritin (40 mg/kg) administration reduced immobility time in tail suspension test (TST) and forced swimming test (FST) without affecting locomotion activity. In CUMS model study, liquiritin (40 mg/kg, once daily for 4 weeks) significantly increased sucrose consumption and body weight of CUMS mice. The behavioral experiment results showed that liquiritin reduced the immobile time of CUMS mice in TST and FST, respectively, and increased the time spent and open arm entries in the elevated plus-maze (EPM) test. Further, the hippocampal superoxide dismutase (SOD) activity was increased in liquiritin-treated group, while malonaldehyde (MDA) decreased. Additionally, the hippocampal cytokines interleukin-18 (IL-18) and interleukin-1 beta (IL-1*β*) contents were reduced in the liquiritin-treated group. Further, liquiritin downregulated the expression of NLRP3 in the hippocampus of CUMS mice rather than TLR4. Besides, NLRP3 inflammasome-associated proteins caspase-1 and ASC were also downregulated. However, liquiritin did not alter the thermal stability of NLRP3 in the cellular thermal shift assay (CETSA), suggesting that its inhibition of NLPR3 was not by direct targeting of NLRP3 protein.

**Conclusions:**

Liquiritin attenuates depression-like behavior of CUMS mice and inhibited cytokines levels triggered by NLRP3 inflammasome, suggesting the antidepressant action is, at least partially, associated with antioxidant stress and inhibition of NLRP3 inflammasome activation.

## 1. Introduction

Accumulating evidence suggests that mitigation of neuroinflammation could be a potential therapeutic approach for depression [1]. Clinical studies have shown that inflammatory cytokines in the central nervous system are significantly elevated, such as IL-1*β* and IL-6, in patients with depression. Recent studies consider that inhibition of NLRP3 inflammasome activation may be a promising strategy for the therapy of depression [2]. Liquiritin, a flavonoid isolated from the root of liquorice, has exhibited various biological activities, such as anti-inflammatory, antiaging, and antitumor activity [3–5]. The neuroprotective activity of liquiritin has attracted considerable attention. Studies have shown that liquiritin could resist the death of cortical neurons induced by glutamate and promote the axon growth of PC12 cells triggered by nerve growth factor [6, 7]. Notably, an early study reported that liquiritin showed antidepressant effect via enhancement of 5-hydroxytryptamine and norepinephrine levels in the hippocampus, hypothalamus, and cortex [8]. In another study, liquiritin also showed antidepressant effects, which might be related to the defense of liquiritin against oxidative stress [9]. Although some evidence confirms the antidepressant effect of liquiritin, it is unclear whether the alleviation of central inflammation is involved in this effect. Therefore, we established an CUMS model to confirm the antidepression effects of liquiritin and explore the effects on cytokine release and NLRP3 inflammasome associate protein expression in the hippocampus.

## 2. Materials and Methods

### 2.1. Drugs and Reagents

**Figure 1:**
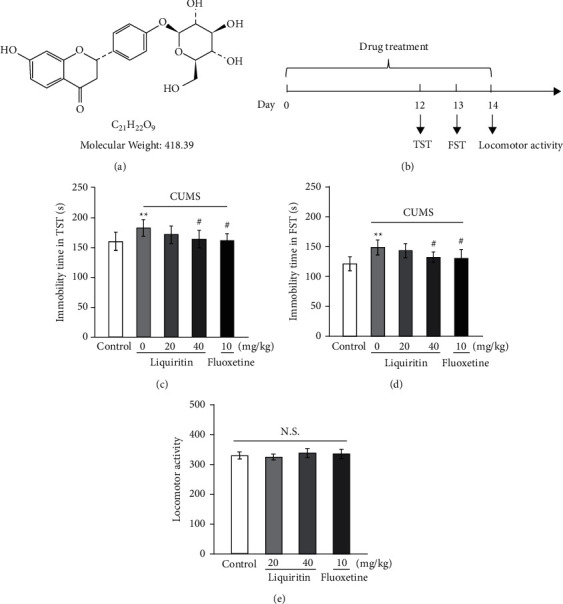
Effects of liquiritin or fluoxetine on desperate behavior in mice. (a) Chemical structure of liquiritin. (b) Schematic diagram of despair experiment. (c) Tail suspension test. (d) Forced swimming test. (e) Locomotor activity. Liquiritin or fluoxetine was administered intragastrically once daily for consecutive 14 days. Data represent means ± SD (*n* = 10). For statistical significance, ^*∗*^*P* < 0.05 and ^∗∗^*P* < 0.01 versus the control group. N.S.: nonsignificant.

Liquiritin (CAS: 551-15-5, molecular formula is shown in Figure 1(a)) and fluoxetine hydrochloride (CAS: 56296-78-7), purity ≥98%, were purchased from Selleck China (Shanghai, China). PC12 cell was purchased from Procell Life Science&Technology Co., Ltd. DMSO (D4540) was got from Sigma-Aldrich (Shanghai) Trading Co., Ltd. Superoxide dismutase (SOD) detection kit (A001-1-2), glutathione peroxidase (GSH-Px) detection kit (A005-1-2), and malondialdehyde (MDA) detection kit (A003-1-2) were all purchased from Nanjing Jiancheng Institute of Biological Engineering (Nanjing, China). BCA protein concentration detection kit (P0012S) and RIPA Lysis Buffer (P0013B) were purchased from Beyotime Biotechnology Co., Ltd. (Shanghai, China). Interleukin-1 beta (IL-1*β*) detection kit (SEKM-0002), interleukin-18 (IL-18) detection kit (SEKM-0019), NLRP3, and TLR4 antibody (K004108P, K003881P) were all purchased from Beijing Solarbio Technology Co., Ltd. (Beijing, China). Caspase-1, pro-caspase-1, IL-1*β*, NEK7, and ASC (ab138483, ab238972, ab283818, ab133514, ab175449) antibodies were obtained from Abcam (Shanghai) Trading Co., Ltd. (Shanghai, China). *β*-Actin antibody (4970) and goat anti-rabbit IgG (HRP polymer) (98164) were purchased from Cell Signaling Technology (Danvers, USA).

### 2.2. Animals

One hundred and twenty male Kunming (KM) mice (18–22 g) were purchased from Yisi Laboratory Animals Company (Changchun, China), whose license number was SCXK (Ji)-2020-0002. Mice were housed at an ambient temperature of 21 ± 2°C and a relative humidity of 45 ± 5% with 12 h light-dark cycle for each day and free access to a standard chow diet and drinking water throughout the experiments.

### 2.3. Experimental Design

**Figure 2:**
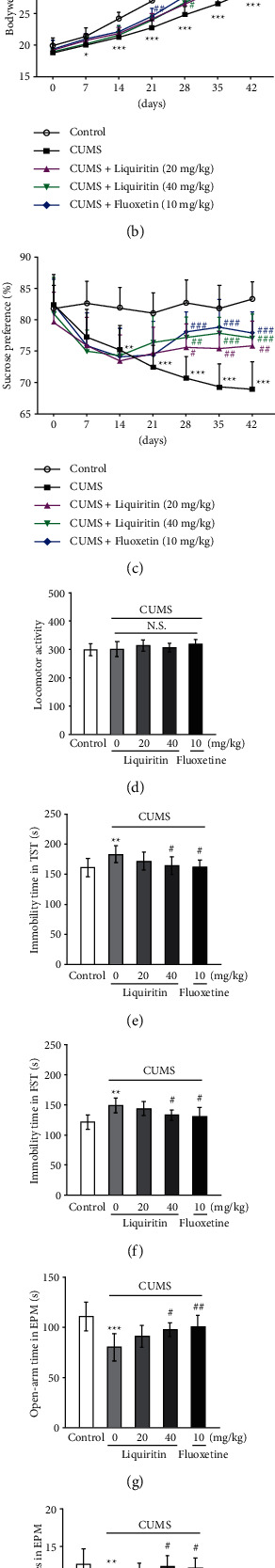
Effects of liquiritin or fluoxetine on depression-like behavior in CUMS mice. (a) CUMS experimental design. (b) Sucrose preference test every 7 days. (c) Bodyweight assessment every 7 days. (d) Locomotor activity in elevated plus-maze. (e) Tail suspension test. (f) Forced swimming test. (g) The total time of mice entered the open-arm in elevated plus-maze. (h) The open-arm entries in elevated plus-maze. Data represent means ± SD (*n* = 10). For statistical significance, ^*∗*^*P* < 0.05, ^*∗∗*^*P* < 0.01, and ^*∗∗∗*^*P* < 0.001 versus the control group; ^#^*P* < 0.05, ^##^*P* < 0.01, and ^###^*P* < 0.001, versus the CUMS group. N.S.: nonsignificant.

Forty mice were used to observe the effect of liquiritin on despair behavior. Mice were randomly assigned to 4 groups (10 for each group), including the control group, liquiritin group (20 mg/kg or 40 mg/kg), and fluoxetine group (positive control, 10 mg/kg). Liquiritin or fluoxetine was injected by intragastric gavage once daily. TST, FST, and locomotor activity tests were performed on days 12, 13, and 14. The process is shown in Figure 1(b). Eighty mice were used to investigate the effect of liquiritin on depressive behavior induced by CUMS. Mice were divided into five groups with sixteen mice per group, including the control group, CUMS group, liquiritin group (20 mg/kg or 40 mg/kg), and fluoxetine group (10 mg/kg). The establishment method of CUMS model was based on the previous research [10] and was modified appropriately. Briefly, stimulus includes fasting (24 h), no water (24 h), clip tail (2 cm from the tip of the tail, 1 min), continuous light for 24 h, cold water swimming (4°C, 5 min), hot water swimming (40°C, 5 min), wet cage (200 ml water per cage, 24 h), tilting cage (45°, 24 h), behavior restriction (1 h), and noise (30 min). Mice were subjected to random stress twice a day for 42 days. After 14 days of CUMS, mice were given saline, liquiritin, or fluoxetine once daily for 28 days. Behavioral experiment was conducted on Day 40 to Day 42. At the end of the experiment, mice were euthanized with sodium pentobarbital (150 mg/kg), and then the hippocampal tissue was isolated quickly and homogenized in an ice bath. Ten samples from each group were used for Western blot and enzyme-linked immunosorbent assay (ELISA); the other six samples were used for immunohistochemistry (IHC). The experimental scheme is shown in Figure 2(a).

### 2.4. Tail Suspension Test (TST)

TST refers to the literature method [11]. Briefly, 1 h after administration, mice were fixed with adhesive tape about 1 cm from the tail tip, hanging 15 cm away from the table. After 2 min of adaptation, 4 min of total immobility time was recorded. The standard of immobility was stopping struggling and hanging vertically.

### 2.5. Forced Swimming Test (FST)

FST has been modified according to the documentary method [12]. In short, 1 h after administration, mice were placed in a Perspex bucket (height 25 cm, diameter 12 cm), filled with water to a height of 10 cm (water temperature was kept at 23 ± 2°C). After 2 min of adaptation, 4 min of total immobility time was recorded. The standard of immobility was stopping struggling, floating upright, or making small movements to keep the head above water.

### 2.6. Locomotor Activity Test

After 1 h of administration, mice were placed in the autonomic activity tester (Chengdu Taimeng Software Co., Ltd., China). After 2 min of adaptation, the locomotor activities of the mice were recorded by camera for 10 min.

### 2.7. Sucrose Preference Test

The test was performed 1 h after administration. During the time, animals were given one bottle of 1% sucrose solution (100 ml) and one bottle of distilled water (100 ml). After 2 h, the consumption of sucrose water and distilled water was measured. We switched the position of the bottles at the midpoint of the test to avoid possible side effects. Sucrose preference is measured as the percentage of sucrose solution consumed relative to total liquid intake. The test was conducted every 7 days.

### 2.8. Elevated Plus-Maze (EPM)

The elevated plus-maze consists of two open arms and two closed arms (arm length 30 cm, arm width 6 cm, elevated height 50 cm) with a central area of 5 × 5 cm. At the beginning of the test, mice were placed on a central platform with their heads towards one open arm. The exploratory behavior was recorded for 5 min, including the open-arm entries and the time spent in each arm. The maze was wiped with 75% alcohol after each animal was done.

### 2.9. Antioxidant Activity and Cytokines Detection

Hippocampus of mice was homogenized in an ice bath and then centrifuged at 12000 rpm for 15 min. The supernatant was separated and stored at 4°C. The activities of SOD and GSH-Px and the content of MDA in the hippocampus were determined by ultraviolet spectrophotometry. The levels of IL-1*β* and IL-18 were measured by ELISA. All operations were carried out according to the kit instructions.

### 2.10. Western Blot Analysis

The total protein of hippocampal tissue was separated by SDS-PAGE gel electrophoresis and transferred onto polyvinylidene fluoride (PVDF) membrane. The dilution ratios of the antibodies were as follows: NLRP3 antibody and TLR4 antibody (1 : 500) and *β*-actin antibody, IL-1*β* antibody, caspase-1 antibody, pro-caspase-1 antibody, IL-1*β* antibody, NEK7 antibody, and ASC antibody (1 : 1000). Then, HRP-labeled secondary antibody (1 : 3000) was added, incubated for 2 h. The gel imaging system was used to detect and photograph, and the strips were analyzed by ImageJ software (Maryland, USA). *β*-Actin was used as an internal reference to calculate relative protein expression.

### 2.11. Immunohistochemistry (IHC)

According to the previous method [13], after the mice were killed, hippocampal tissues were quickly removed and soaked overnight in 10% paraformaldehyde at 4°C to make paraffin sections. Rabbit TLR4 antibody (1 : 100) and rabbit NLRP3 antibody (1 : 100) were used for IHC detection after dewaxing treatment. The sections were washed and incubated with the secondary antibody (1 : 3000). Subsequently, an image of the hippocampal CA3 region was obtained using an optical microscope and analyzed by ImageJ software.

### 2.12. Cellular Thermal Shift Assay (CETSA)

According to the documentary method [14], 2 × 10^6^ PC12 cells and 8 ml RPMI-1640 were separately added in *φ*100 mm dishes. After incubation for 24 h, liquiritin was added at a final concentration of 200 *μ*m, and DMSO was used as a control. With a continuous incubation for 2 h, cells were collected. After centrifugation, cell lysates were aliquoted and individually heated to the indicated temperatures (ranging from 25°C to 65°C) for 3 min and then cooled at room temperature for 3 min, repeating it for 3 cycles. Insoluble proteins were separated by centrifugation, and then a Western blot analysis was used to detect the changes in the NLRP3 protein.

### 2.13. Statistical Analysis

Statistical analysis was performed using GraphPad Prism 8.0 software (California, USA). The experimental data were expressed as means ± standard deviation (SD). One-way ANOVA analysis was used to compare the sample means between groups. *P* < 0.05 indicates that the difference is statistically significant.

## 3. Results

### 3.1. Liquiritin Alleviated the Desperate State and Depression-like Behavior in Mice

The desperate behavior test was performed to initially evaluate the antidepressant effect of liquiritin in naive mice. As shown in Figures 1(c) and 1(e), after 14 days of intragastric administration with liquiritin (40 mg/kg), the immobile time of TST and FST was reduced, respectively, compared with the control group. To rule out the interference of liquiritin on central excitability, a locomotion activity test was carried out. The results showed that liquiritin showed no significant effect on locomotion activity.

CUMS model was established to further investigate the antidepression effect of liquiritin. The CUMS model was thought to imitate the main symptoms of depression and has been widely used to explore the pathogenesis of depression [15]. Reduced sucrose consumption is regarded as a key indicator in animals [16]. We found that sucrose consumption and body weight decreased significantly from day 14 in the liquiritin-treated group (Figures 2(b) and 2(c)). On days 40 and 41, TST and FST were undertaken. Liquiritin (40 mg/kg) reduced immobile time in CUMS mice, respectively (Figures 2(e) and 2(f)). On day 42, we performed an elevated plus-maze, widely used to evaluate anxiety and depression in rodents [17]. We observed a significant increase in the time spent and times of entering the open arms in the liquiritin group (Figures 2(g) and 2(h)). However, there was no significant difference in locomotive activity between the liquiritin and CUMS groups (Figure 2(d)). In all tests, the positive drug fluoxetine showed significant antidepression effects in mice, consistent with previous reports [18]. All the data above indicated that liquiritin attenuated the depression-like behavior of CUMS mice.

### 3.2. Liquiritin Enhanced the Antioxidant Capacity of CUMS Mice

**Figure 3:**
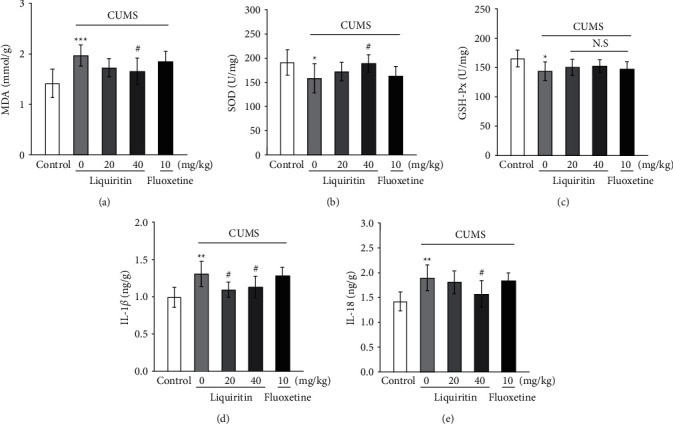
Effects of liquiritin or fluoxetine on antioxidant activity and inflammatory cytokines in hippocampus tissues. (a) MDA levels. (b) SOD activity. (c) GSH-Px content. (d) IL-1*β* content measured by ELISA. (e) IL-18 content measured by ELISA. Data represent means ± SD (*n* = 6). For statistical significance, ^*∗*^*P* < 0.05, ^*∗∗*^*P* < 0.01, and ^*∗∗∗*^*P* < 0.001 versus the control group; ^#^*P* < 0.05 versus the CUMS group. N.S.: nonsignificant.

A series of studies have shown that acute and chronic stress trigger oxidative stress damage in brain regions, which is highly associated with depression [19]. It has been confirmed that the depressive behavior of rats is related to the increased level of lipid peroxidation metabolite MDA, suggesting that the harmful effects of CUMS on the mood and behavior of rats are, at least partly, mediated by oxidation [20]. Here, we found that the MDA content of mice increased significantly in CUMS group. In the liquiritin- (40 mg/kg) treated group, SOD activity was increased, and MDA content was decreased in the hippocampus of CUMS mice (Figures 3(a) and 3(b)). However, there was no significant difference in GSH-Px activity among all groups (Figure 3(c)). The above results indicate that liquiritin attenuated oxidative stress and improved the antioxidant capacity of CUMS mice.

### 3.3. Liquiritin Decreased the Levels of IL-18 and IL-1*β* in the Hippocampus of CUMS Mice and Inhibited the Expression of NLRP3 Inflammasome Activation

**Figure 4:**
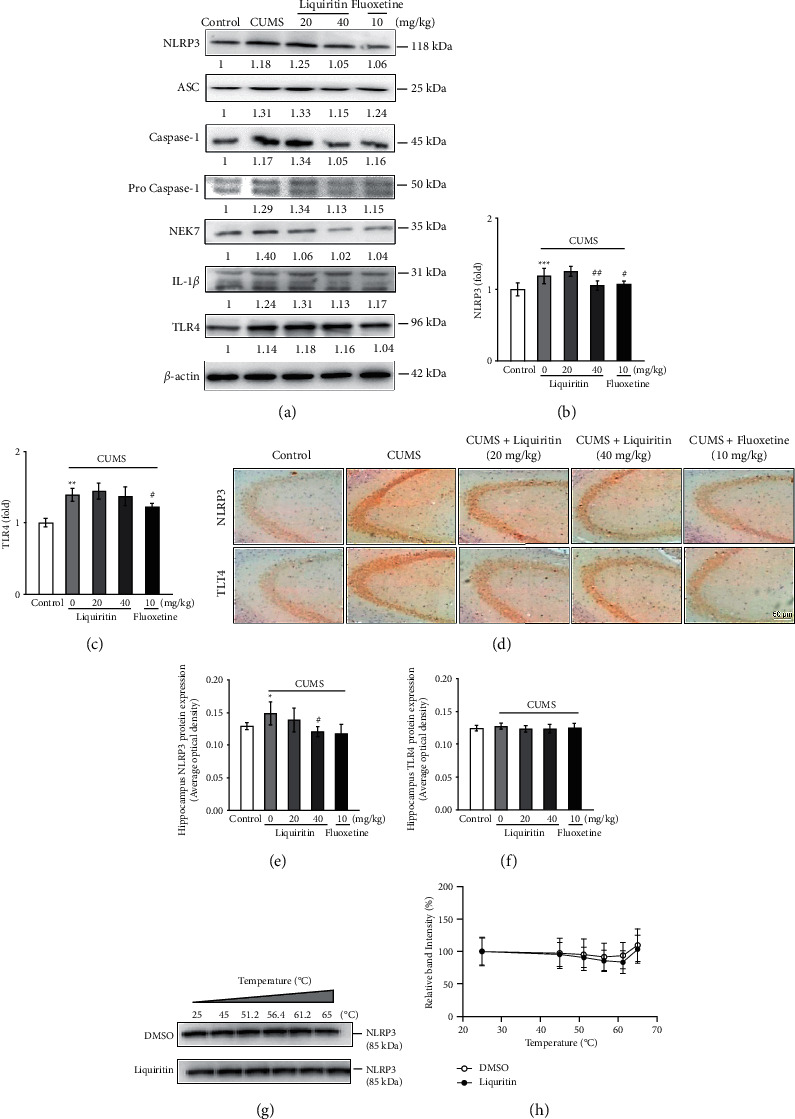
Effects of liquiritin or fluoxetine on the expression of inflammatory proteins and the interactions with proteins. (a) NLRP3 inflammasome-associated protein expression in the hippocampus. *β*-Actin was used as an internal reference. (b, c) Quantification of NLRP3 and TLR4 protein expression by Western blot (*n* = 3). (d) Expression of NLRP3 and TLR4 in hippocampal CA3 region measured by IHC. Positive cells are represented as brown spots. (e, f) Quantification of NLRP3 and TLR4 expression by IHC (*n* = 3). (g, h) CETSA and quantification. Quantification of both Western blot and IHC was performed using ImageJ software. Data represent means ± SD (*n* = 3). For statistical significance, ^*∗*^*P* < 0.05, ^*∗∗*^*P* < 0.01, and ^*∗∗∗*^*P* < 0.001 versus control group; ^#^*P* < 0.05 and ^##^*P* < 0.01 versus the CUMS group.

It has been found recently that IL-1*β* and IL-18 play an important role in depression. In our study, we found that the levels of IL-1*β* and IL-18 in the hippocampus of CUMS mice were higher than those of the control group (Figures 3(d) and 3(e)). Liquiritin (40 mg/kg) reduced the contents of IL-18 and IL-1*β* in the hippocampus of CUMS mice, respectively. In order to clarify the pathway by which liquiritin inhibits inflammatory response, we detected the protein expression of NLRP3 and Toll-like receptor 4 (TLR4), which was considered to contribute to the production and secretion of the mature IL-1*β*. The results showed that liquiritin (40 mg/kg) downregulated the expression of NLRP3 in the hippocampus of CUMS mice (Figures 4(a) and 4(b)). By contrast, it showed no significant effect on TLR4 expression (Figures 4(a) and 4(c)), and the IHC data confirmed this result (Figures 4(d)–4(f)). Additionally, we found that liquiritin (40 mg/kg) downregulated the expression of NLRP3 inflammasome-associated proteins, including ASC, caspase-1, pro-caspase-1, IL-1*β,* and NEK7 in the hippocampus of CUMS mice (Figure 4(a)). These results suggest that liquiritin inhibited the release of cytokines IL-18 and IL-1*β* by inhibiting the NLRP3 inflammasome-mediated inflammatory activity, rather than TLR4 mediated pathway.

The CETSA was used to evaluate the binding ability of drugs to proteins [21]. After treating PC12 cells with liquiritin or DMSO, cell lysates were heated at temperature ranging from 25°C to 65°C. The western blot was conducted using a specific antibody for NLRP3. The results suggested liquiritin did not alter the thermal stability of the NLRP3 protein compared to that of DMSO, implying that liquiritin might not bind to NLPR3 by direct action (Figures 4(g) and 4(h)).

## 4. Discussion

In this study, we mainly found that chronic administration of liquiritin improved depression-like behavior of CUMS mice and inhibited inflammasome activation and downstream inflammatory cytokines levels in the hippocampus. As the thermal stability of NLRP3 protein was not altered by liquiritin, liquiritin might inhibit NLRP3 inflammasome activation by acting indirectly through a potential target.

The main symptoms of depression include low mood, reduced activity, and aversion to activities [22]. The CUMS model successfully simulated these core symptoms of depression, and we therefore employed it to evaluate the antidepressant effects of liquiritin. A significant reduction in the immobility time of TST and FST was found, as well as an increase in sucrose consumption, suggesting that liquiritin enhanced the happiness and social exploration interest of CUMS mice.

Studies have demonstrated that the pathogenesis of depression is related to the inflammatory process in the central nervous system, with changes in the release of cytokines, increased oxidative stress, and regulation of the glutathione system [8, 9]. Moreover, clinical observations have identified that depression is associated with elevated levels of inflammatory cytokines in the blood, while anticentral inflammatory strategies were effective in improving depressive symptoms [23]. Depression exhibited increases in IL-1*β* and IL-18 levels associated with activation of NLRP3 inflammasome [24]. NLRP3 inflammasome consists of three important components: the NLRP3 protein as a sensor, apoptosis-associated speck-like protein containing CARD (ASC) as an adaptor, and caspase-1 as an effector [25]. In the normal state, ASC is located in the nucleus, while NLRP3 and pro-caspase-1 are present in the cytoplasm. When cells are stimulated by pathogens, NLRP3 recruit and bind to ASC to form an activated NLRP3 inflammasome complex, consequently resulting in pro-caspase-1 activation [26]. Caspase-1 cleaves the precursors of IL-1*β* and IL-18 to produce the corresponding mature cytokines [27]. NIMA-related kinase 7 (NEK7) is widely expressed in the brain, which is related to the initiation of mitosis, cell cycle progression, and mitotic progression [28]. Studies have found that NEK7 regulated the activation of NLRP3, which played an important role in the inflammatory cascade of macrophages [29]. Here, we show that liquiritin (40 mg/kg) inhibited the protein expression of NLRP3 inflammasome components in the hippocampal region by western blot and IHC assay, accompanied by significantly lower levels of IL-1*β* and IL-18 as well. The inhibition of NLRP3 inflammasome may be responsible for the antidepressant properties of liquiritin. We also performed CETSA to verify whether there is a direct binding of liquiritin to NLPR3. It was found liquiritin failed to affect the thermal stability of NLRP3 protein, indicating no direct binding of liquiritin to NLRP3 protein. We, therefore, suggest that liquiritin may indirectly inhibit the activation of NLRP3 inflammasome through other potential upstream targets.

In addition to the NLRP3 inflammasome, TLR4 may also be involved in the inhibition of inflammatory cytokines released by liquiritin [30]. When stress is sustained or severe, TLR4 in the cell membrane enhances the expression levels of nuclear factor-*κ*B (NF-*κ*B) by both MyD88-dependent and non-MyD88-dependent pathways, which in turn promotes the formation and release of proinflammatory substances such as IL-1*β* and tumor necrosis factor *α* (TNF-*α*) [31]. Herein, we found that liquiritin had little effect on TLR4 protein expression, suggesting that liquiritin inhibition of IL-1*β* and IL-18 may be associated with NLRP3 rather than TLR4.

Liquiritin is a natural compound that has been shown to possess anti-inflammatory effect, antioxidant activity, and neuroprotective and antitumor benefits [32–35]. The inhibitory effect of liquiritin on IL-1*β* was also present in other studies. It has been shown to improve rheumatoid arthritis by reducing VEGF expression in IL-1*β*-induced RA-FLS cells by blocking MAPK signaling pathway [34]. Although liquiritin was not found to downregulate TLR4 expression in the hippocampus of CUMS mice in this study, it has been found to do so in studies of cyclophosphamide-induced hepatotoxicity [33]. In line with most studies, we confirmed the antioxidant effect of liquiritin as evidenced by an increase in hippocampal SOD levels and a decrease in MDA content in CUMS mice. However, to our knowledge, the inhibitory effect of liquiritin on the NLRP3 inflammasome is reported here for the first time. Collectively, our study demonstrates for the first time that liquiritin reduces the release of central cytokines by inhibiting NLRP3 inflammasome. As an intensively studied anti-inflammatory natural compound, liquiritin holds promise for development as an adjuvant antidepressant.

## Data Availability

The datasets used and analyzed during the current study are available from the corresponding author on reasonable request.

## Supplementary Materials

Supplementary Materials Figure S1: HPLC analysis of liquiritin. Figure S2: license for the use of laboratory animals.
