# Corrigendum to “Liquiritin Alleviates Depression-Like Behavior in CUMS Mice by Inhibiting Oxidative Stress and NLRP3 Inflammasome in Hippocampus”

**DOI:** 10.1155/ecam/9768027

**Published:** 2025-07-02

C. Liu, D. Yuan, C. Zhang, Y. Tao, Y. Meng, M. Jin, W. Song, B. Wang, and L. Wei, “Liquiritin Alleviates Depression-Like Behavior in CUMS Mice by Inhibiting Oxidative Stress and NLRP3 Inflammasome in Hippocampus,” *Evidence-Based Complementary and Alternative Medicine* 2022 (2022), https://doi.org/10.1155/2022/7558825.

In the article titled “Liquiritin Alleviates Depression-Like Behavior in CUMS Mice by Inhibiting Oxidative Stress and NLRP3 Inflammasome in Hippocampus,” Figure 1c/d is inadvertently duplicated with Figure 2 e/f.  Figure 1 should be corrected as follows:

We apologize for this error.

## Figures and Tables

**Figure 1:**
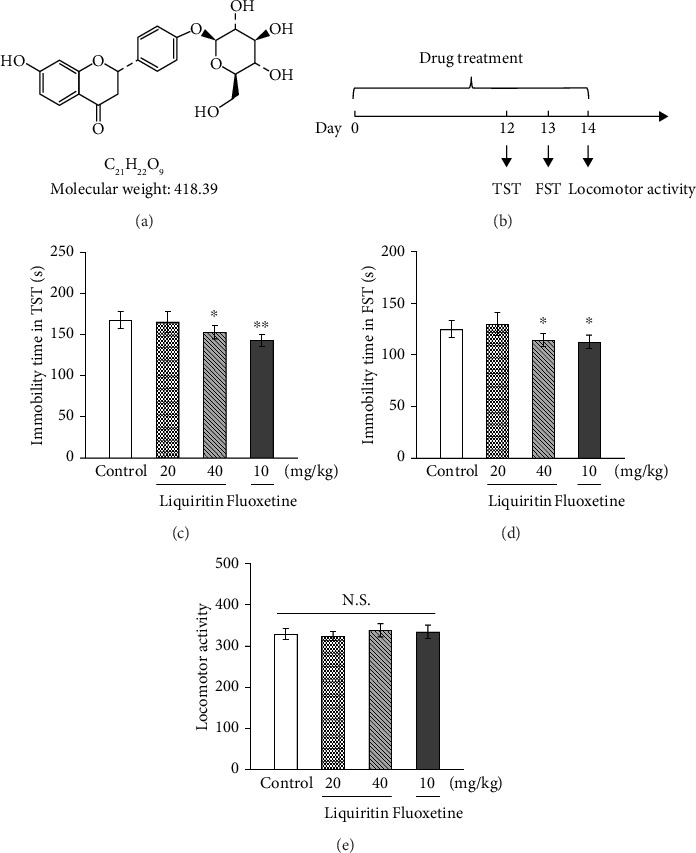
Effects of liquiritin or fluoxetine on desperate behavior in mice. (a) Chemical structure of liquiritin. (b) Schematic diagram of despair experiment. (c) Tail suspension test. (d) Forced swimming test. (e) Locomotor activity. Liquiritin or fluoxetine was administered intragastrically once daily for consecutive 14 days. Data represent means ± SD (*n* = 10). For statistical significance, ^∗^*p* < 0.05 and ^∗∗^*p* < 0.01 versus the control group. N.S.: nonsignificant.

