# Supplementary material for: Liquiritin Alleviates Depression-Like Behavior in CUMS Mice by Inhibiting Oxidative Stress and NLRP3 Inflammasome in Hippocampus
Source: Evid Based Complement Alternat Med. 2022 Jan 11;2022:7558825. doi: 10.1155/2022/7558825 (PMC8767389; doi:10.1155/2022/7558825)
Supplement: Supplementary Materials — Figure S1: HPLC analysis of liquiritin. Figure S2: license for the use of laboratory animals. [file 7558825.f1.doc]

**Supplementary Information**

**Liquiritin alleviates depression-like behavior in CUMS mice by inhibiting oxidative stress and NLRP3 inflammasome in hippocampus**

Chang Liu,^1†^ Dai Yuan,^1†^ Chi Zhang,^2^ Ye Tao,^3^ Ying Meng,^1^ Mengli Jin,^1^ Wu Song,^1*^ Bingmei Wang,^1*^ and Lin Wei^1*^

^1^ Clinical Medical College of Changchun University of Chinese Medicine, Changchun, 130117, China

^2^ School of Basic Medicine, Changchun University of Chinese Medicine, Changchun, 130117, China

^3^ Affiliated Hospital of Changchun University of Chinese Medicine, Changchun, 130021, China

†These authors contributed equally.

Correspondence should be addressed to Wu Song; five841110@126.com, Bingmei Wang; bingmeiwang1970@163.com and Lin Wei; lynnw2013@sina.com

**Figure S1. HPLC analysis of liquiritin**

**Figure S2. License for the use of laboratory animals**

**Figure S1.**

**
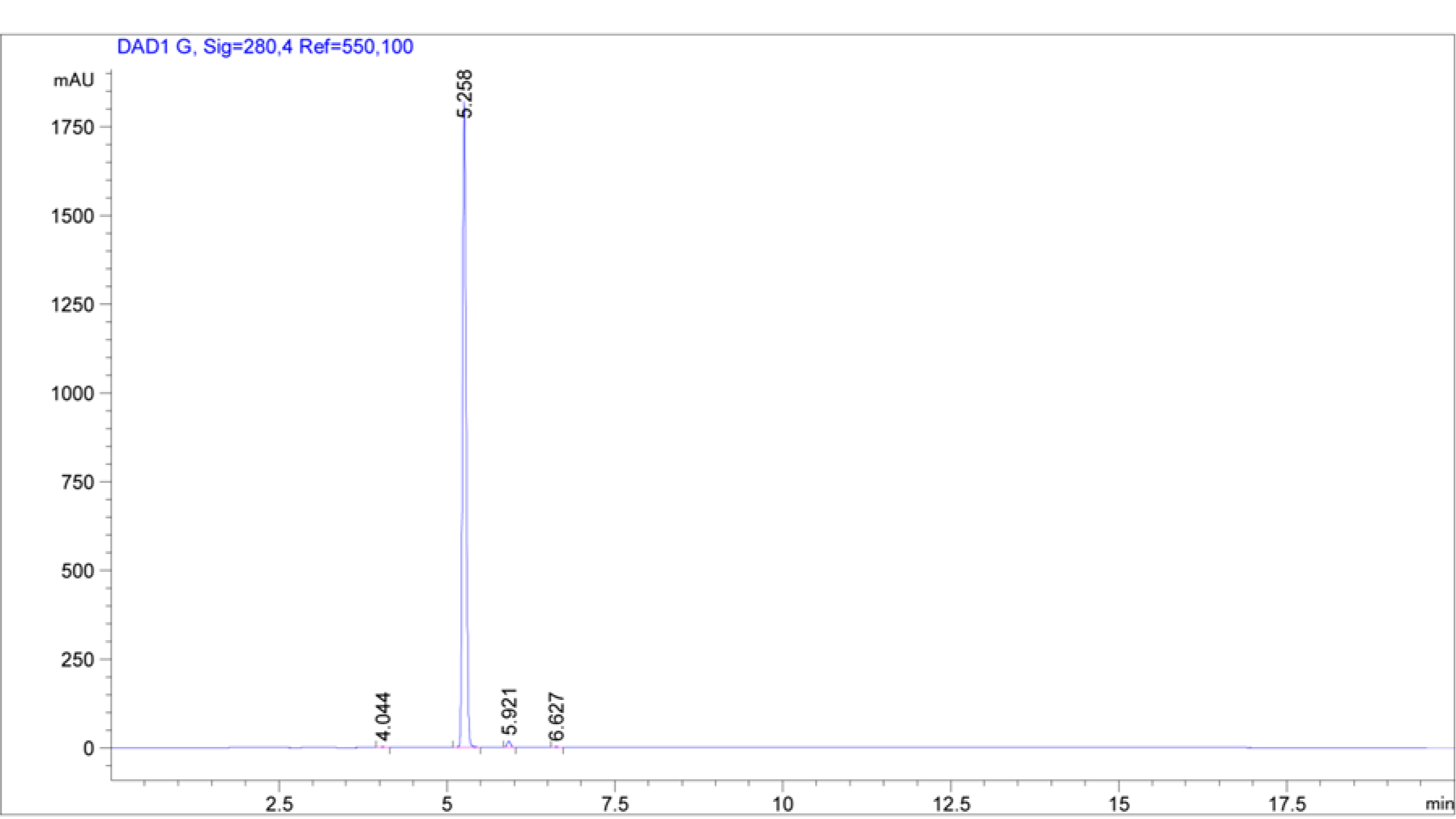
**

**Figure S1. HPLC analysis of liquiritin.**

**Figure S2.**

**
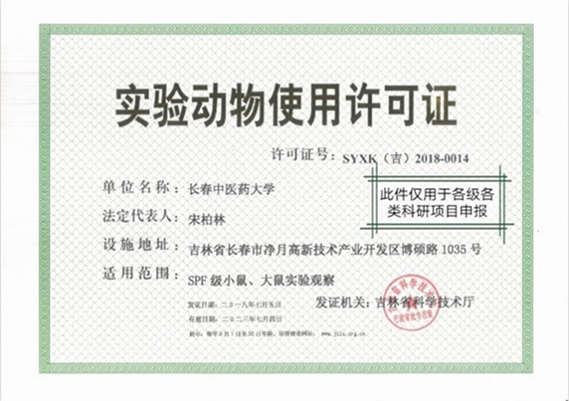
**

**Figure S2. License for the use of laboratory animals**.
